# Transarticular screw fixation using neuronavigation: Technique

**DOI:** 10.4103/0019-5413.36994

**Published:** 2007

**Authors:** Srinivas Dwarakanath, Ashish Suri, Bhavani Shankar Sharma

**Affiliations:** Department of Neurosurgery, Neurosciences Center, All India Institute of Medical Sciences, New Delhi, India

**Keywords:** C1-C2, neuronavigation, transarticular screw

## Abstract

**Background:**

Transarticular screw placement needs highly accurate imaging. We assess the efficacy and accuracy of C1-C2 transarticular screw fixation using neuronavigation and also cast a technical note on the procedure.

**Materials and Methods:**

This study included a total of nine patients who underwent transarticular screw fixation using the neuronavigation system. A total of 15 screws were placed. All patients underwent postoperative CT scan with 3-Dimensional (3-D) reconstruction to check for the accuracy of implantation.

**Results:**

One patient had encroachment of the transverse foramen but there was no vertebral artery injury. There were no clinical complications or adverse sequelae.

**Conclusion:**

Neuronavigation is extremely helpful in C1-C2 transarticular screw fixation and gives excellent accuracy.

Conventional techniques in spinal surgery require the surgeon to infer the location, dimension and trajectory of the screw based on the visible and palpable anatomic landmarks.^1^ Conventional fluoroscopy assisted the surgeon in these. However, various factors such as anatomic variability, distorted or deformed anatomy, inability to identify or lack of anatomic landmarks and poor imaging or uniplanar imaging with conventional fluoroscopy have limited or sometimes thwarted the surgeon from proceeding with the surgery or resulted in complications.^1^ Though intraoperative monitoring techniques such as somatosensory evoked potentials (SSEP), electromyography (EMG) have been used as adjuncts, they are not reliable and alert the surgeon only after damage has occurred.^1^

The usefulness of spinal navigation has been supported by the available literature. Conventional techniques have reported screw error placement from 0-50% and neurological sequelae of 5% while in studies where image guidance was used, there has been a significant decrease in spinal screw misplacement (0-14%) with no clinical sequelae.^1^

This study has been designed in order to study the efficacy of intraoperative navigation in C1-C2 transarticular screw fixation.

## MATERIALS AND METHODS

The present study included all patients (n=9) admitted from March 2002 to July 2006 who underwent C1-C2 transarticular screw placement under image guidance. Preoperatively, the patient's CT was performed according to the set protocol. The images were transferred to the workstation via the picture archiving and communication system (PACS) and a 3-D image reformatted along with images in the sagittal, coronal and axial planes. Preoperative planning and virtual surgery were performed; entry and target points were selected and the trajectory was reconstructed along the given path. The screws of appropriate length and width were selected and the relation to specific neural, vascular and osseus structures computed [Figures 1A–1C]. Discrete anatomic points were selected on the segments to be instrumented. These points were then surgically exposed [Figure 2]. The dynamic reference system was attached to the spinous process of C2 and the previously marked points registered through paired point registration and if necessary surface to surface merge. Image-patient registration fusion was attained. To verify the accuracy, the probe was placed on various landmarks on the exposed spine, laminae and facets and coincided with the respective images. Then the screw was placed along the desired trajectory under image guidance. Virtual fluoroscopy was performed using the FluoroNav System, which included planning and navigation at that level.

**Figure 1A:**
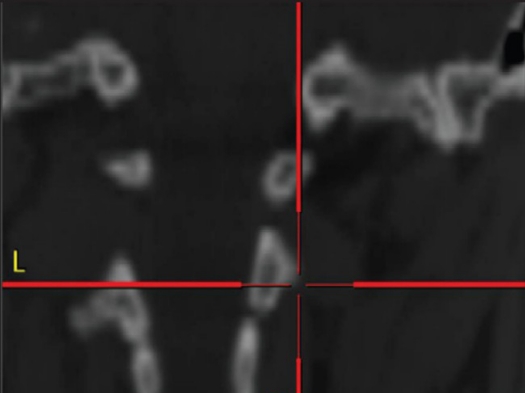
Entry point of the screw on C2 (Right-sided transarticular screw)

**Figure 1B:**
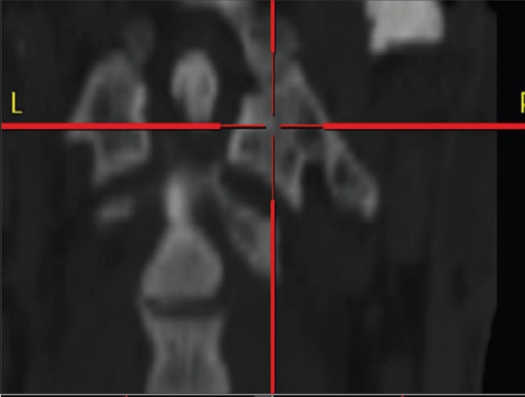
Target point of the screw on C1 (Right-sided transarticular screw)

**Figure 1C:**
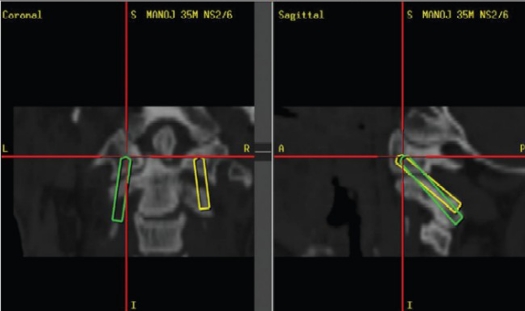
Virtual trajectory of the screw on either side (Green leftsided: Yellow right-sided)

**Figure 2:**
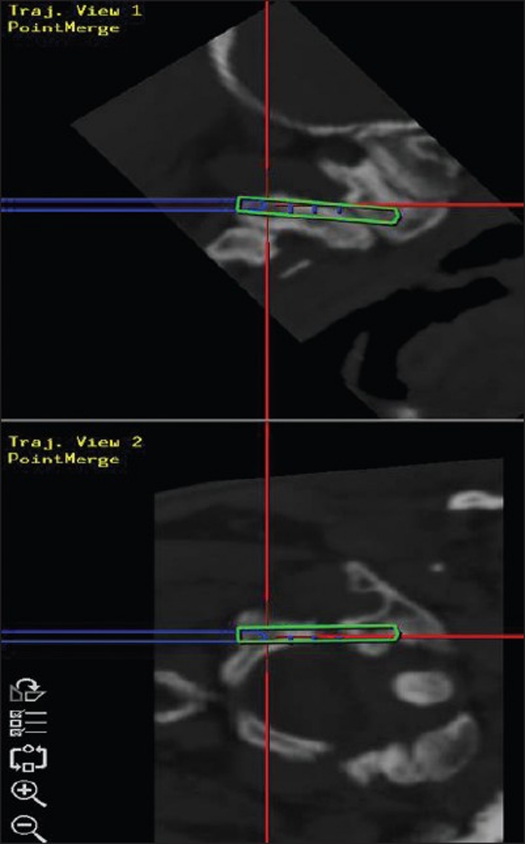
Intraoperative navigation (blue line shows the path taken intraoperatively)

The demographic characters of the patients were studied. When the patient underwent image-guided spinal instrumentation (IGS), the approach, accuracy of navigation, details of instrumentation, time taken and specific utility of neuronavigation intraoperative course and any complications were noted. Postoperative imaging was performed to locate the position of the screw in relation to the bony landmarks, neural and vascular structures.

## RESULTS

Nine consecutive patients who underwent image-guided C1-C2 transarticular screw fixation (n=15), from March 2002 to July 2006 [Table 1] are presented. There were seven males and two females in the study; their ages ranged from 16-56 years.

**Table 1:** Spinal image-guided surgery: spectrum, indications and outcome

Case Number	Indication	Procedure planned	Screws placed	Screws	Problems	Outcome	Clinical outcome
1	Traumatic AAD*	C1C2 Trans articular screw + Gallies fixation	2	2	-	Screws *in situ*	Uneventful
2	Traumatic AAD*	C1C2 Trans articular screw + Gallies fixation	2	2	-	One screw encroaching vertebral foramen	Uneventful
3	Congenital AAD*	C1C2 Trans articular screw	2	2	-	Screws *in situ*	Uneventful
4	Congenital AAD*	C1C2 Trans articular screw	2	1	Failure of the IGS• •	Screws *in situ*	Uneventful
5	Rheumatic AAD*	C1C2 Trans articular screw	2	2	-	Screws *in situ*	Uneventful
6	Rheumatic AAD*	Gallies fixation	2	-	Bone very brittle Procedure abandoned	-	-
7	AAD* + Fracture Odontoid	TOO† + PD• + and posterior fixation using C1 C2 Transarticular screws	2	2	-	Screws *in situ*	Uneventful
8	AAD*+ Fracture C2	C1C2 Transarticular screw fixation and C3 lateral mass plating	2	2	-	Screws *in situ*	Uneventful
9	Traumatic AAD*	C1C2 Trans articular screw	2	2	-	Screws *in situ*	Uneventful

* AAD - Atlantoaxial Dislocation,

† TOO - Transoral Odontoidectomy,

‡ PD - Posterior decompression,

• • IGS - Image-guided spinal instrumentation

**Figure 3:**
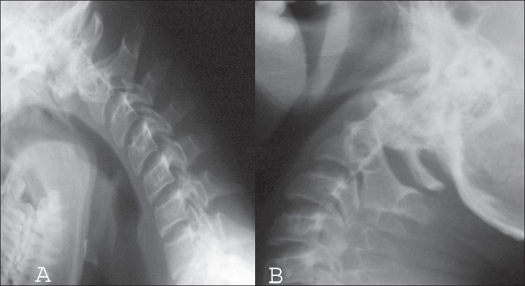
Lateral X-rays of cervical spine (flexion - extension) shows a reducible AAD (3A-Flexion, 3B Extension)

**Figure 4:**
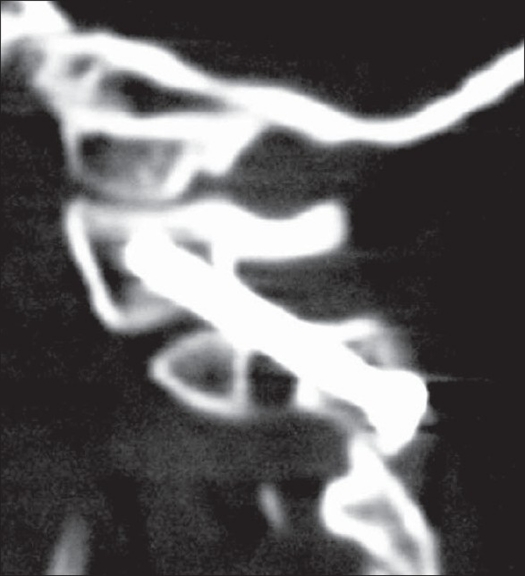
Postoperative CT scan of the patient shown in figure 3 with reconstruction showing the well positioned screws on either side

The clinical details, treatment strategies, technical problems encountered and outcome are summarized in Table 1. Postoperative CT scan with 3-D reconstruction revealed highly accurate screw placement [Figures 3,4]. Among the 15 only one screw had encroached upon the transverse foramen.

Spinal instrumentation under image guidance was performed with no mortality or morbidity. The procedure was abandoned for technical reasons after placement of one screw in a patient, while in another the procedure was abandoned as the rheumatoid bone was too brittle and fractured with screw placement (despite appropriate planning and navigation). None of the patients developed any postoperative neurovascular deficit.

## DISCUSSION

Our study consisted of nine patients who underwent C1-C2 transarticular screw fixation. IGS provides an excellent 3-D anatomical reconstruction, thus helping immensely in the planning and virtual screw placement, guiding the trajectory of the intraoperative approach and accurate placement of screws.

Bloch *et al* assessed the accuracy of image guidance for atlantoaxial transarticular screw placement in cadaveric spine specimens.^2^ They concluded that image guidance improves the safety of screw placement and potentially allows this procedure to be performed in patients excluded otherwise due to the inaccuracy of conventional techniques. In an American association of neurological surgeons/ Congress of neurological surgeons (AANS/CNS) survey, of the 2492 C1-C2 transarticular screw placements, the vertebral artery injury rate was 2.2%/screw and the risk of neurological deficit due to this was 0.1%/screw.^3^ Haid *et al* used a combination of C1-C2 transarticular screws along with a posterior interspinous construct in 75 patients (141 screws) with atlantoaxial instability under fluoroscopy.^4^ Postoperative complications (8%) included wound infection in two patients and transient suboccipital numbness in four. Osseous fusion was evident in 72 patients (96%). No instances of vertebral artery injury, dural injury or errant screw placement were seen. Campanelli *et al* used transarticular screws (fluoroscopic guidance) in seven geriatric patients (13 screws).^5^ Intraoperative complications were limited to one screw (7.5%) in which the vertebral artery was injured. Weidner *et al* used image-guided surgery for C1-C2 transarticular screw fixation comparing postoperative screw position in a nonrandomized prospective cohort (37 patients) with a historic control group (78 patients) in which fluoroscopic guidance was used alone.^6^ They concluded that image-guided surgery reduced but did not eliminate the risk of screw misplacement and the overall surgical time was not increased. Similarly Acosta *et al* used neuronavigation during C1-C2 transarticular screw fixation in 20 patients (36 screws). They were able to achieve an accuracy of 92 % (32/36). Normal C1-C2 alignment was achieved in 17 of 20 (85%) patients.^7^ Wigfield *et al* inserted 84 C1-C2 transarticular screws in 46 patients without any neurovascular injury.^8^ In our study, while we had a technical failure in a single screw placement, 15 screws were placed successfully with an accuracy rate of 93%.

## CONCLUSIONS

The present study although a small series, highlights the application of image-guided transarticular screw fixation. Spinal image guidance is very useful in planning, intraoperative navigation and screw placement and minimizes error and should soon become the gold standard for this procedure.
